# Study and Optimization of The Necessary Conditions for The Sensitive Determination of The Lead Ion by a Modified Carbon Paste Electrode in Environmental Water Samples

**Published:** 2018

**Authors:** Masoumeh Ghalkhani, Fatemeh Ghelichkhania, Fatemeh Ghorbani-Bidkorbeh

**Affiliations:** a *Department of Chemistry, Faculty of Science, Shahid Rajaee Teacher Training University, Lavizan, Tehran, Iran.*; b *Department of Pharmaceutics, School of Pharmacy, Shahid Beheshti University of Medical Sciences, Tehran, Iran.*

**Keywords:** Carbon paste, Voltammetry, Determination, Lead, Bismuth, Carbon nanotube

## Abstract

Rapid and facile preparation of the cheap modified electrode materials is an important parameter in development of the efficient electrochemical sensor for industrial scale production and mass-market usage. In the present work, the carbon paste electrode modified with multi-walled carbon nanotubes (MWCNTs) was prepared for sensitive determination of lead (Pb) ion in the presence of bismuth (Bi) ion due to synergetic effect of carbon nano-materials and Bi on the voltammetric response. Investigations showed that in presence of Bi ion degassing of the test solution is not needed. Supporting electrolyte, required Bi concentration and accumulation time and potential were optimized for differential pulsed anodic stripping voltammetric tests. Under optimized conditions, modified carbon paste electrode showed sensitive voltammetric response in the range of 0.1-10 μM toward Pb ion. Moreover, efficiency of the developed method was evaluated for the determination of Pb ion in several water samples. The obtained recovery results of 92-97 % revealed good agreement between responses of this sensor and common spectroscopies techniques for the Pb ion analysis. The optimized system has high efficiency in reproducibility and repeatability of the results and applicability for the analysis of the various environmental water samples.

## Introduction

Regarding the versatility, rapidness, and low cost, electrochemical sensors are considered as appropriate tool for continuous monitoring and determination of various analytes in different fields such as agricultural, pharmaceutical, food and environmental industries and furthermore in medical diagnosis (1, 2). It is worth mentioning that the voltammetric response recorded by an electrochemical sensor is dependent on the surface area of the working electrode (3, 4).

On the other hand, researchers are trying to develop portable and miniaturized measuring systems, especially for on-site measurements of trace amounts of various analytes in real samples such as biological fluids (whole blood, urine, plasma, serum and saliva). However, application of the very small electrodes reduces and loses the sensitivity of the common electrochemical measurements. Therefore, it is necessary to increase the effective surface area per unit volume of the electrode using suitable modifiers, which leads to significant enhancement of voltammetric response and the analysis sensitivity (5-7). Moreover, the stability, repeatability, and reproducibility of the measurement results are very important factors that should be considered in development of sensory systems.

Regarding to the benefits of nanotechnology, nanostructured materials can be used to modify and increase the electrode surface. Up to the present, different materials on a nano scale were employed for electrode surface modification such as metal nanoparticles (8, 9), nano metal oxides (10, 11), carbon based nanomaterials such as fullerene (12), carbon nanotubes (13-15), graphene (16, 17), and carbon nanoparticles (18-20). Different studies have shown that in addition to high thermal, chemical and mechanical stability, carbon nanotubes have excellent electrical conductivity and; on the other hand, have a very high active surface that is very suitable for modifying the electrode surface (13). The most importantly is the possibility of the physical or chemical modification of the carbon nanotubes surface, which doubles their effectiveness as the electrode modifiers.

Today, due to the large extent use of heavy and toxic metals in industrial processes (such as plating, batteries and paints), environmental contamination by heavy and toxic metal ions such as lead has been a serious problem (21, 22). Since, Pb^2+^ ion is a non-biodegradable toxic heavy metal; its accumulation problem in the environment becomes more serious day-by-day (23). Also, the Pb^2+^ ion accumulation in the human body can lead to various injuries in various tissues of the body such as the kidneys, liver, central nervous system and bones (24). Therefore, the development of a cheap, sensitive and selective sensor system for accurate and fast measurement of toxic heavy metal ions such as Pb^2+^ is important (25). Various modified electrodes have been developed to measure Pb^2+^ ions in diverse samples (26-32). 

In the anodic stripping voltammetric technique, at first metal ions are adsorbed on the electrode surface from the bulk solution by physical or chemical interactions, and subsequently reduced to the atomic metal by applying the potential step (33, 34). Then, in the anodic sweep potential, deposited metal is oxidized and removed from the electrode surface resulting anodic current proportional to deposited metal ion in the accumulation step (2, 32). In order to achieve a better response, it is necessary to use a suitable electrode substrate that can be simply modified with suitable modifier and can effectively improve the voltammetric response. Between different substrates, carbon paste is a good option due to its wide range of working potential, especially in the positive potentials, low background current, convenient and inexpensive production of primary materials, simple and fast surface renewal, effective surface, and bulk modification. Highly efficient with good repeatability modified electrode can easily be prepared conveniently by hand mixing of suitable percentage of carbon nanotubes with carbon paste.

In the present work, using functionalized carbon nanotubes with acidic functional groups, a modified carbon paste electrode (CNT/CPE) was constructed, which was then used to study the sensitive measurement of Pb^2+^ ion in the presence of bismuth ion.

## Experimental

*Chemical and reagents*

carbon nanotubes (CNTs) obtained by catalytic chemical vapor deposition (CVD) technique (purity ˃ 95 %) having outer diameter of 10-20 nm, inner diameter of 5-10 nm and tube length of 0.5-200 nm were purchased from Nanostructured & Amorphous Materials (Houston, TX, USA). Graphite powder (˂ 20 μm) purchased from Aldrich co. was employed for carbon paste preparation. All other used materials were of analytical reagent grade and purchased from Merck.

*Apparatus*

Voltammetric experiments were performed using a potentiostat/galvanostat SAMA 500, electroanalyzer system, I. R. Iran. A three-electrode cell was employed, including a carbon paste working electrode (d = 3.0 mm, unmodified or modified), an Ag/AgCl (saturated KCl) reference electrode and a Pt wire auxiliary electrode. Image of scanning electron microscopy (SEM) was obtained using a Holland Philips XL30 scanning electron microscope. Electrochemical impedance spectroscopy (EIS) tests were carried out by a Zahner elektrik IM6 and booster (Module PP240) potentiostat/galvanostat between 0.1 Hz to 100 kHz using a 5 mV rms sinusoidal modulation in KCl (0.1 M) solution containing 1 mM of K_4_Fe (CN)_6_ and 1 mM K_3_Fe(CN)_6_ (1:1) mixture.

*The CNTs Functionalization*

Amount of 1.0 g of the purchased CNTs was transferred into a round bottom volumetric flask containing 10 mL 1:3 (v/v) mixture solution of concentrated nitric acid and sulfuric acid was put on the magnetic stirrer for 20 min to homogenize its suspension. Then, its content was refluxed for 2 h and after cooling down the final mixture was diluted with deionized water (DW) and centrifuged to separate the functionalized CNTs. Repeatedly; functionalized CNTs were dispersed in DW and centrifuged until the pH of the CNTs dispersion reaches to about 7. Finally, collected functionalized CNTs were dried at 70 °C in a vacuum oven. As a result, open-end, carboxylated CNTs with hydrophilic surfaces were prepared.

*Preparation of the carbon paste electrodes (CPEs)*

Unmodified carbon paste was prepared by complete mixing of graphite powder with suitable amount of silicone oil (about 75:25 w/w). CNT modified carbon paste was prepared by mixing 1000 mg of unmodified carbon paste with 200 mg functionalized CNTs. To better homogenize the resulted paste, 5 mL of dichloromethane was added to the paste and completely mixed, then modified paste was left in the room temperature for 24 h to remove the solvent by self-vaporization. At last, obtained carbon paste was used for construction of the modified electrode named as CNT/CPE. A portion of the prepared pastes was packed firmly into the cavity (3.0 mm diameter) of a Teflon tube. The electric contact was achieved by pushing a copper rode inside of the electrode body. Prior to voltammetric experiments, the CPE surface was polished on a soft paper and then thoroughly washed with deionized water. If it was needed the electrode surface was renewed simply by mechanical polishing on a soft paper. 

## Results and Discussions

*Characterization*

**Figure 1:**
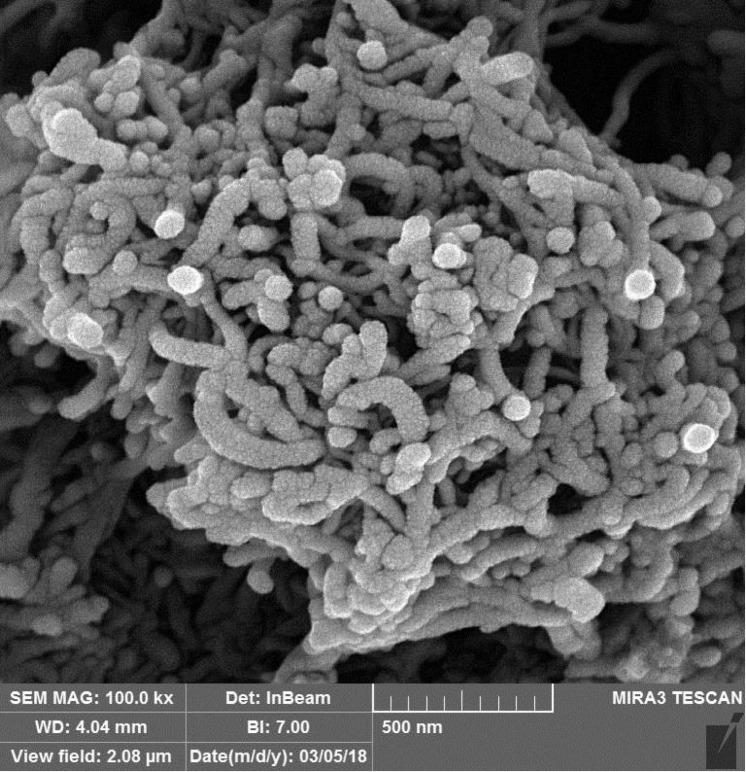
SEM image of the carboxylated CNTs

Figure 1 shows the microscopic structure of functionalized CNTs taken by SEM technique in which high effective surface area of the CNTs is observable. CPE modification with functionalized nanostructures led to significant enhancement of the electrode surface area that regarding to the presence of acidic functional groups on the CNTs surface causes the increment of the voltammetric response sensitivity because of the high affinity towards the effective adsorption of Pb^2+^ ions onto the electrode surface.

The properties of the charge transfer at the interface of the electrode surface and electrolyte was evaluated by EIS. In EIS diagram there are two parts of the semicircle section and linear part. The semicircle section is related to the resistance to charge transfer that is mainly related to the limitation of the electron transfer kinetic across the interface of the Helmholtz layer and the CNT/CPE surface. The linear section is related to the electrde process restricted by the mass transfer. EIS diagrams recorded at frequency between 0.1 Hz – 100 kHz. Regarding the diameter of the semicircle part of the nyquest plot, the bare CPE shows high resistance to electron transfre while the obtained amount for CNT/CPE was very smaller. Since the CNT/CPE has low resistanse to electron transfer, it can provide an effective electron transfer path at the interface of the electrolyte and the electrode surface. Comparison of the ion conductivity of the CNT/CPE with unmodified CPE revealed a significant increase of the ion conductivity of CPE containg CNTs that led to a considrable enhancement of the electrochemical efficiency of the modified CPE.

*Evaluation of the voltammetric response of the bare CPE to Pb* ^2+^ *ion*

**Figure 2:**
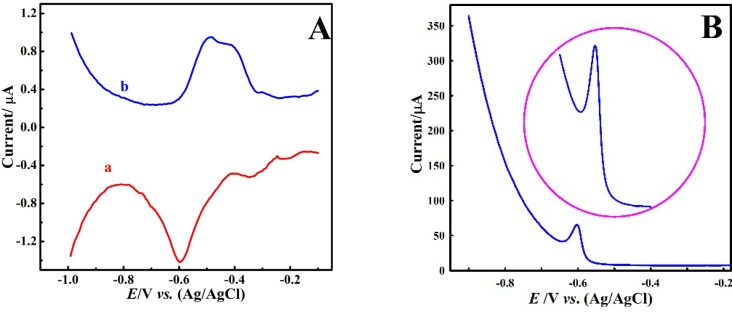
(A) DPV (a) and DPASV (b) of Pb^2+^ (1.0 μM) at bare CPE in HAC (pH 4),*E*_d_ = -1 V, t_d_ = 120 s; (B) DPASV of Pb^2+^ (20 μM) at bare CPE in HNO_3_ (0.1 M),*E*_d_: deposition potential, t_d_ = deposition time, DPASV= Differential pulse anodic striping voltammetry

At first, differential pulse and differential pulse anodic striping voltammetric, (DPV) and (DPASV), respectively, response of the bare CPE (BCPE) were recorded in 0.1 M acetate buffer solution of pH 4 containing 1 μM Pb^2+^ ion. Figure 2A shows the obtained voltammograms that reveals weak and undesirable response because their peaks are wide with small currents. This indicates that the employed analysis conditions were not suitable for Pb^2+^ ion determination. Therefore, low sensitivity and undesirable responses were obtained. In the next step, electrolyte was changed and nitric acid (0.1 M) was used. The DPASVs in Figure 2B show the improvement in the voltammetric response compared to acetate buffer electrolyte. Therefore, in subsequent experiments, nitric acid solution was used as the background electrolyte.

*Comparison of the voltammetric response of Pb* ^2+^ *ion at the BCPE and CNT/CPE*

**Figure 3:**
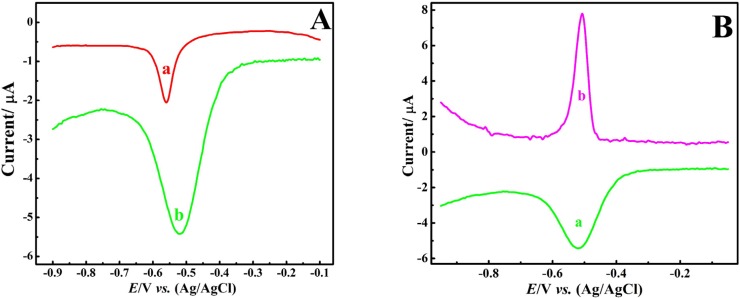
(A) DPV of Pb^2+^ (1 μM) at (a) bare CPE and (b) CNT/CPE; (B) DPV (a) *vs.* DPASV (b) of Pb^2+^ (1 μM) at CNT/CPE in HNO_3_

Figure 3A compares the DPV response of BCPE and CNT/CPE to 1 μM Pb^2+^ ion in nitric acid (0.1 M) electrolyte solution. Comparison of the response of Figure 3A with Figure 2A indicates a significant improvement in the response, both in terms of the peak current and the voltammograms shape and the decrease of peak width, which again indicates the positive effect of nitric acid electrolyte on the sensor output. Moreover, the comparison of the (a) and (b) voltammograms of Figure 3A obviously shows the significant enhancement of the electrochemical response to Pb^2+^ ion in the presence of CNTs, albeit minor increase in peak width could be an undesirable factor in the sensor selectivity in complex samples.

Then, DPASV of the Pb^2+^ ion was recorded at the CNT/CPE and compared with DPV response, Figure 3B. Obtained results revealed effective improvement of voltammetric response, both in terms of the peak current enhancement and the peak width reduction.

**Figure 4:**
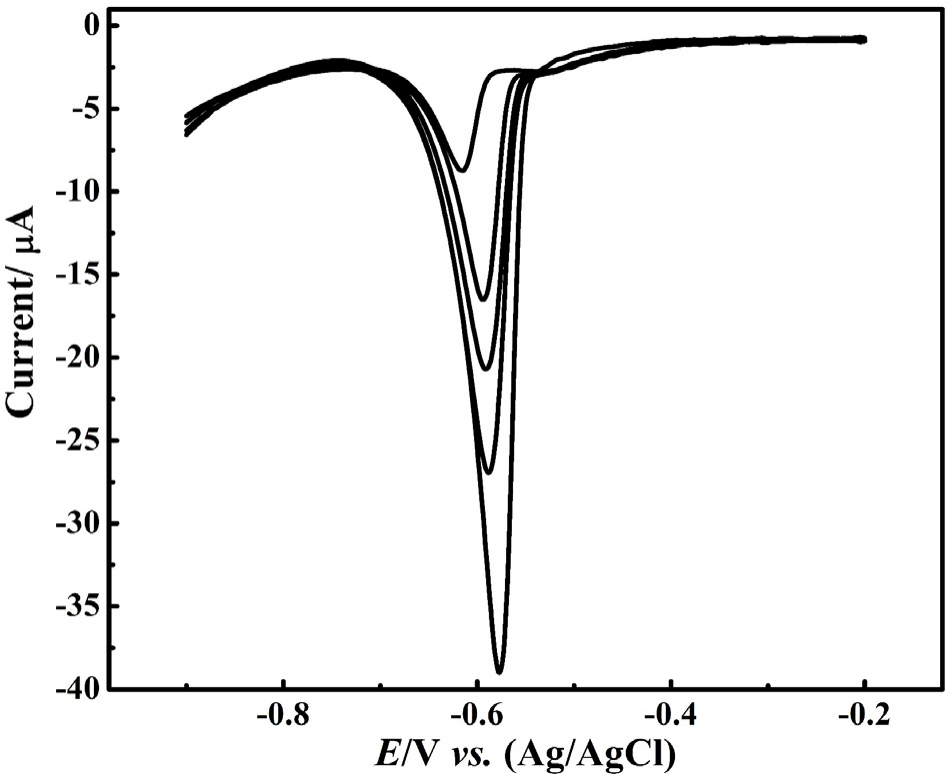
DPV calibration of Pb2+ in HNO (0.1 M) at CNT-CPE; up to down: 20 – 100 μM

Quantification determination of the Pb^2+^ ion was performed by DPV method at the CNT/CPE, Figure 4 The Resulted voltammograms showed a linear variation of the peak current with concentration in the range of 20 – 100 μM. Notably, even though in lower concentration, the Pb^2+^ ion reduction peak was observed but it did not follow linear relationship of the current-concentration. In addition, the electrode surface saturation occurred at higher concentration and the peak current variation was inappreciable while the peak width was increased at higher concentrations.

*Optimization of the analysis condition*

**Figure 5:**
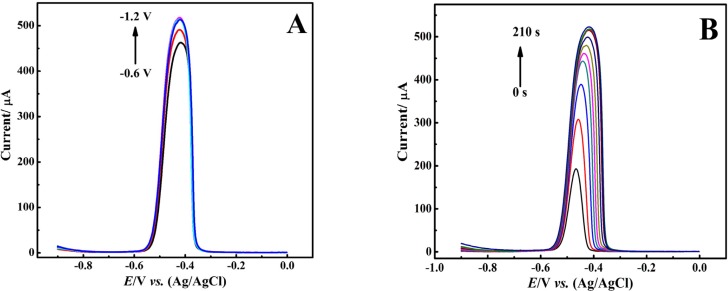
DPASVs of 100 μM Pb^2+^ in HNO_3_ (0.1 M) at CNT-CPE under various (A) deposition potential (*E*_d_) and (B) deposition time (t_d_)

Considering better response, we chose DPASV technique for further experiments. In stripping technique two important parameters, potential and time of catodic reduction, should be considered. Here, at first the effect of these parameters was examined on the DPASV of Pb^2+^ ion. Figure 5A shows the striping voltammograms of 100 μM Pb^2+^ ion in nitric acid (0.1 M) electrolyte under electro-deposition time (t_d_) of 160 s with different electro-deposition potentials (*E*_d_) (from -0.6 to -1.2 V). Obtained results indicated that the peak current of the voltammograms was increased following the negative shift of *E*_d_ from -0.6 to -1.2 V revealing more reduction of Pb^2+^ ions under a certain t_d_ on the surface of the modified electrode.

However, the peak current did not significantly changed with applying *E*_d_ more negative than -0.9 V, while it caused the peaks broadening. This observation might be due to increased thickness of the Pb film electrodeposited on the CNT/CPE surface causing peak broadening in the anodic stripping step. As a result, *E*_d_ = -0.9 V was chosen as optimum electro-deposition potential.

**Table 1:** Response stability of the sensor for 1.0 µM Pb2+ determination in successive days

**Standard addition to Tap water**	**1** **st ** **Day**	**2** **nd ** **Day**	**3** **rd ** **Day**	**4** **th ** **Day**	**5** **th ** **Day**
DPASVs peak current of Pb2+ (µA) a	20.41 ± 0.94	20.32 ± 0.84	20.02 ± 1.23	19.98 ± 0.74	19.82 ± 1.81
b Stability of peak current (%)	100	99.56	98.09	97.89	97.1

a Average of three replicate measurements (rounded).

b The results have been rounded.

**Figure 6:**
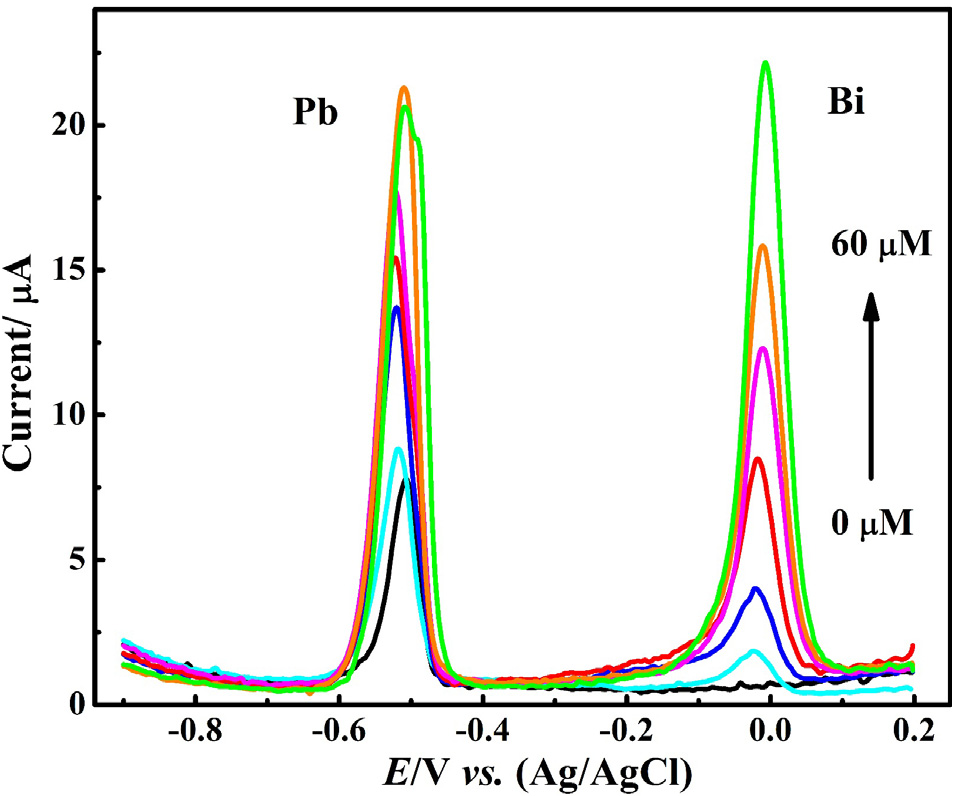
DPASVs of Pb2+ (1 μM) in presence of various concentration of Bi3+ in HNO (0.1 M) at CNT/CPE, *E *= -0.9 V, t = 60 s

**Figure 7:**
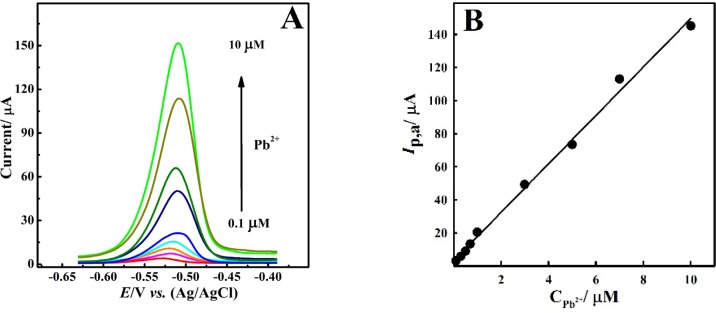
(A) DPASVs for different Pb^2+^ ion concentrations in the range of (down to up) 0.1 - 10 μM, at the CNT/CPE and 50 μM Bi^3+^ in 0.1 M HNO_3_, *E*_d_ = -0.9 V and t_d_= 60 s. (B) Corresponding linear calibration curve of *I*_p,a_ *vs.* Pb^2+^ ion concentration

Furthermore, we evaluated the effect of t_d_ on the stripping voltammograms (Figure 5B). Recorded voltammograms showed that increasing t_d_ from 5 to 60 s resulted in enhancement of stripping peak current along with the observation of sharp voltammograms with small peak width. With further increase of deposition time up to 160 seconds, although we still saw an increase in peak current; however, peak sharpness was lowered and voltammograms were broadened. As a result, applying t_d_ more than 160 seconds leads to the distinct broadening in the shape of voltammograms without remarkable enhancement in peak current. It should be noted here that in the early stages of increasing t_d_, more lead ions are reduced and deposited on the CNT/CPE surface, so that the resulting stripping voltammograms show higher peak currents. On the other hand, with further increment of t_d_ from 60 to 160 seconds, the electrode surface moves towards saturation, and the thickness of the deposited lead layer is rising. Therefore, along with the enhancement in peak current, the peak broadening occurs too. 

However, according to the obtained results, it can be concluded that the total saturation of the CNT/CPE surface occurs at about t_d_ = 160 s. Therefore, a further increase in t_d_ only leads to an increase in the thickness of the lead layer deposited on the surface of the CNT/CPE and thus it causes the broadening of the stripping peaks. Consequently, considering the measurement speed, the higher current and the reduction of the voltammogram width to achieve increased sensitivity and selectivity, t_d_ = 60 was considered for further experiments.

Apart from the problem of limiting the linear range of quantitative measurement using CNT/CPE, due to electrochemical interference of the reduction of dissolved oxygen in the solution in Pb^2+^ determination and the proximity of their reduction peak potentials, deoxygenation step is needed before voltammetry measurements. However, it causes the measurement process to be longer and more complicated and it is required to use an inert gas capsule such as nitrogen or argon. Therefore, according to the results of previously published research papers, we have developed a fast method not requiring deoxygenation of the solution. 

The reports have demonstrated that the electrode surface modification with bismuth metal leads to the increment of the overvoltage and the slowdown of the oxidation-reduction reaction in the electrolyte and as a result, the oxygen gas interruptions in the voltammetric measurements are eliminated. In the present work, the addition of bismuth nitrate to the sample solution was used for further studies. Notably, Bi^3+^ ion is reduced in less negative potential than Pb^2+^ ion.

Figure 6 displays DPASVs of nitric acid (0.1 M) electrolyte solution containing 1 μM Pb^2+^ ion and various values of Bi^3+^ ion recorded using CNT/CPE. In the absence of Bi^3+^ ion, a relatively sharp voltammogram was observed in lead ion solution deoxygenation by nitrogen gas. After adding Bi^3+^ ion to the sample solution the peak current of Pb^2+^ ion was increased and bismuth ion peak was appeared at more positive potential than Pb^2+^ ion peak. Notably, by increasing the concentration of Bi^3+^ ion in the presence of a constant concentration of Pb^2+^ ion, the peak currents of both observed oxidation peaks were improved. Increasing the Bi^3+^ ion concentration up to 50 μm resulted in an effective increase in peak current of Pb^2+^ ion. 

However, further increase in the concentration of Bi^3+^ ion not only did not improve the response of Pb^2+^ ion, but also was associated with the broadening and decreasing of current of DPASV of Pb^2+^ ion. This observation can be related to an increase in the thickness of the metal film formed on the CNT/CPE surface and the occupancy of -most active surface areas by Bi^3+^ ions that are more easily reduced than Pb^2+^ ions. To achieve better results, the Bi^3+^ ion concentration was adjusted to 50 μM as optimal value for furthers investigations.

*Determination of Pb* ^2+^ *concentration by developed procedure and evaluation of the repeatability, stability and reproducibility of CNT/CPE*

Figure 7A shows the DPASVs of Pb^2+^ ion recorded in the presence of 50 μM Bi^3+^ ion in nitric acid electrolyte. Recorded voltammograms presented a wide linear calibration plot between peak current and Pb^2+^ ion concentration in the linear range of 0.1 to 10 μM with a detection limit of 78 nM (Figure 7B) according to Equ. (1).

*I*_p,a _(μA( = 14.642 C_Pb_^2+^ (μM) + 3.159 (R^2^ = 0.9953, n=3)                               Equ.1

The applicability of the developed sensor depends upon two main factors, the reproducibility and repeatability of its measurements. The repeatability of the CNT/CPE response toward Pb^2+^ ion was evaluated with four consecutive measurements of 0.5 and 1.0 μM Pb^2+^ ion, for which resulted value of RSD less than 3% indicated good precision of the CNT/CPE. Regarding that, the amount of the modified carbon paste that once prepared can be employed several times to fabricate many electrodes, the similar electrodes can be fabricated, and hence it can effectively improve the reproducibility of the sensory results. On the other hand, because the prepared carbon paste can be stored in a container for a long time without change, even about one year, the reproducibility of the electrodes produced at different times is also great.

Finally, stability of CNT/CPEs was examined by daily voltammetric measurement of constant concentration of Pb^2+^ ion in drinking tap water sample of Hashtgerd and storing the CNT/CPEs in the laboratory environment within 5 days. The quality of employed water sample was within the acceptable limits set by the World Health Organization, but the hardness levels were relatively high. 

The total dissolved solid, chemical oxygen demand, pH, electric conductivity, heavy metal ions content, including Pb²⁺, in tap water were within the standard limits. Obtained results confirmed maintenance of 97.1% of the initial peak current of DPASVs after 5 days indicating long time stability of the prepared electrodes.

## Conclusion

In the present work, we prepared carbon paste electrode modified with CNTs, investigated and optimized the effective factors on their voltammetric response towards Pb^2+^ ion. The obtained results indicated that the simultaneous addition of Bi^3+^ ion in the measured solution improves sensitivity and selectivity of the sensor toward Pb^2+^ ion, and it renders the possibility of the voltammetric measurements in negative potential without the need for deoxygenation of the solution. Performed experiments revealed that background electrolyte has an effective role on the sensitivity and shape of the recorded voltammograms in Pb^2+^ ion solution and nitric acid was chosen as the most suitable electrolyte. Investigations showed that the presence of Bi^3+^ ion in solution and carbon nanostructures on the electrode surface contribute to an effective reduction of lead ions onto the electrode surface during the electro-deposition step. Therefore, it causes response enhancement and peak width decreasing of the DPASVs of Pb^2+^ ion in the stripping step. Pb^2+^ ion determination in the different water samples applying developed procedure under optimized conditions showed acceptable efficiency of the CNT/CPE for rapid, accuracy, and sensitive quantification of Pb^2+^ concentration.
